# Supplementary material for: Therapeutic targeting nudix hydrolase 1 creates a MYC-driven metabolic vulnerability
Source: Nat Commun. 2024 Mar 16;15:2377. doi: 10.1038/s41467-024-46572-6 (PMC10944511; doi:10.1038/s41467-024-46572-6)
Supplement: Supplementary file 1 — Supplementary Info [file 41467_2024_46572_MOESM1_ESM.pdf]

Supplementary information

Therapeutic targeting nudix hydrolase 1 creates a MYC-driven metabolic vulnerability

Minhui Ye, Yingzhe Fang, Lu Chen, Zemin Song, Qing Bao, Fei Wang, Hao Huang, Jin Xu, Ziwen Wang, Ruijing Xiao, Meng Han, Song Gao, Hudan Liu, Baishan Jiang, Guoliang Qing

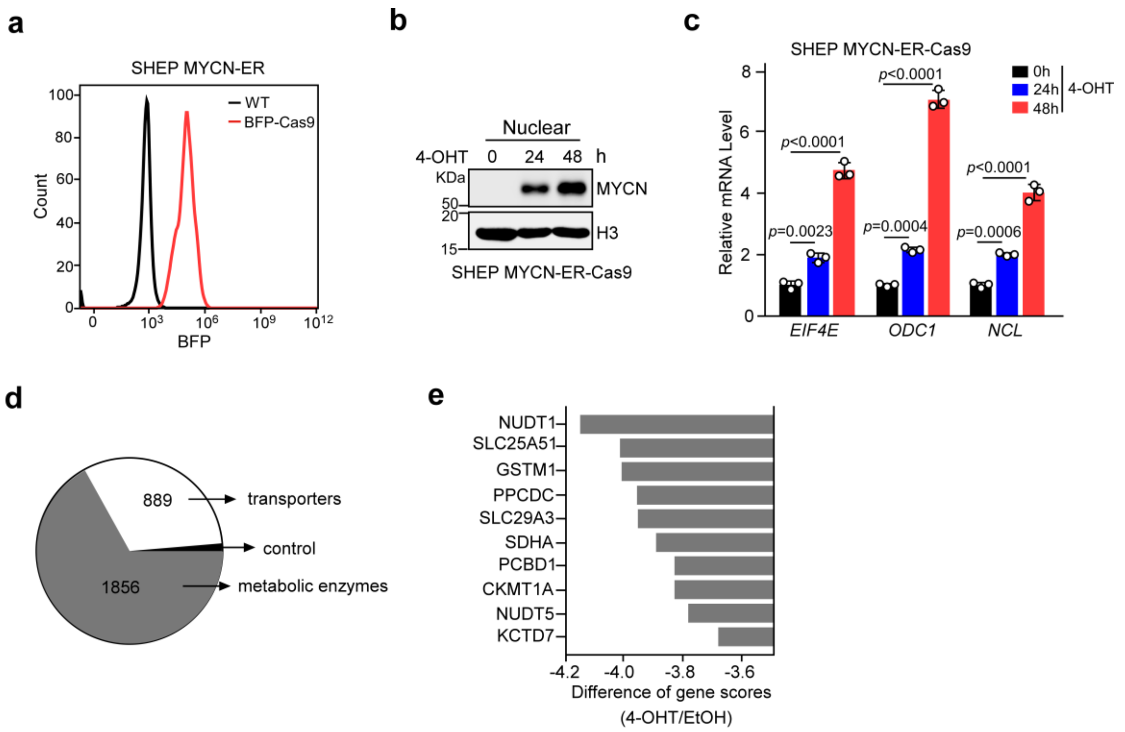

f

GO: ID	Term name	*P*-value
GO:0042262	DNA protection	2.69e-5
GO:0006629	Lipid metabolic process	1.11e-3
GO:0006730	One-carbon metabolic process	4.87e-3
GO:0045454	Cell redox homeostasis	7.92e-3
GO:0006750	Glutathione biosynthetic process	8.34e-2
GO:0055085	Transmembrane transport	3.24e-2
GO:0055086	Nucleobase-containing small molecule metabolic process	3.66e-2
GO:0090461	Glutamate homeostasis	4.56e-2

g

GO: ID	Term name	*P*-value
GO:0006486	Protein glycosylation	2.19e-8
GO:0015718	heparin biosynthetic process	3.33e-5
GO:0019318	Hexose metabolic process	1.28e-3
GO:0008202	Steroid metabolic process	3.71e-3
GO:0046513	Ceramide biosynthetic process	1.39e-2
GO:0000271	Polysaccharide biosynthetic process	1.62e-2
GO:1901137	Carbohydrate derivative biosynthetic process	4.27e-2

Supplementary Figure 1. Construction and validation of sgRNA screen system.
a Flow cytometry analysis of BFP^+ SHEP MYCN-ER cells after Cas9 virus infection for 3 days. **b** Immunoblot of nuclear MYCN in SHEP MYCN-ER-Cas9 cells upon 4-OHT treatment (200 nM) for indicated times. Histone 3 (H3) was used as an internal control. The experiments were independently repeated three times with similar results. **c** qPCR analysis of representative MYC targets in SHEP MYCN-ER-Cas9 cells upon 4-OHT treatment (200 nM) for indicated times. Data shown as average ± SD from technical triplicates. **d** Categories of genes included in the metabolism-focused sgRNA library. **e** Top 10 genes scoring as most differentially required for SHEP MYCN-ER cells upon 4-OHT treatment. **f** GO enrichment analysis of 189 depleted genes with a significant log_2 fold change ($p<0.05$). **g** GO enrichment analysis of the 111 accumulated genes with a significant log_2 fold change ($p<0.05$). **a-c**: these experiments were independently repeated three times with similar results. Statistical significance was determined by one-way ANOVA (**c**).

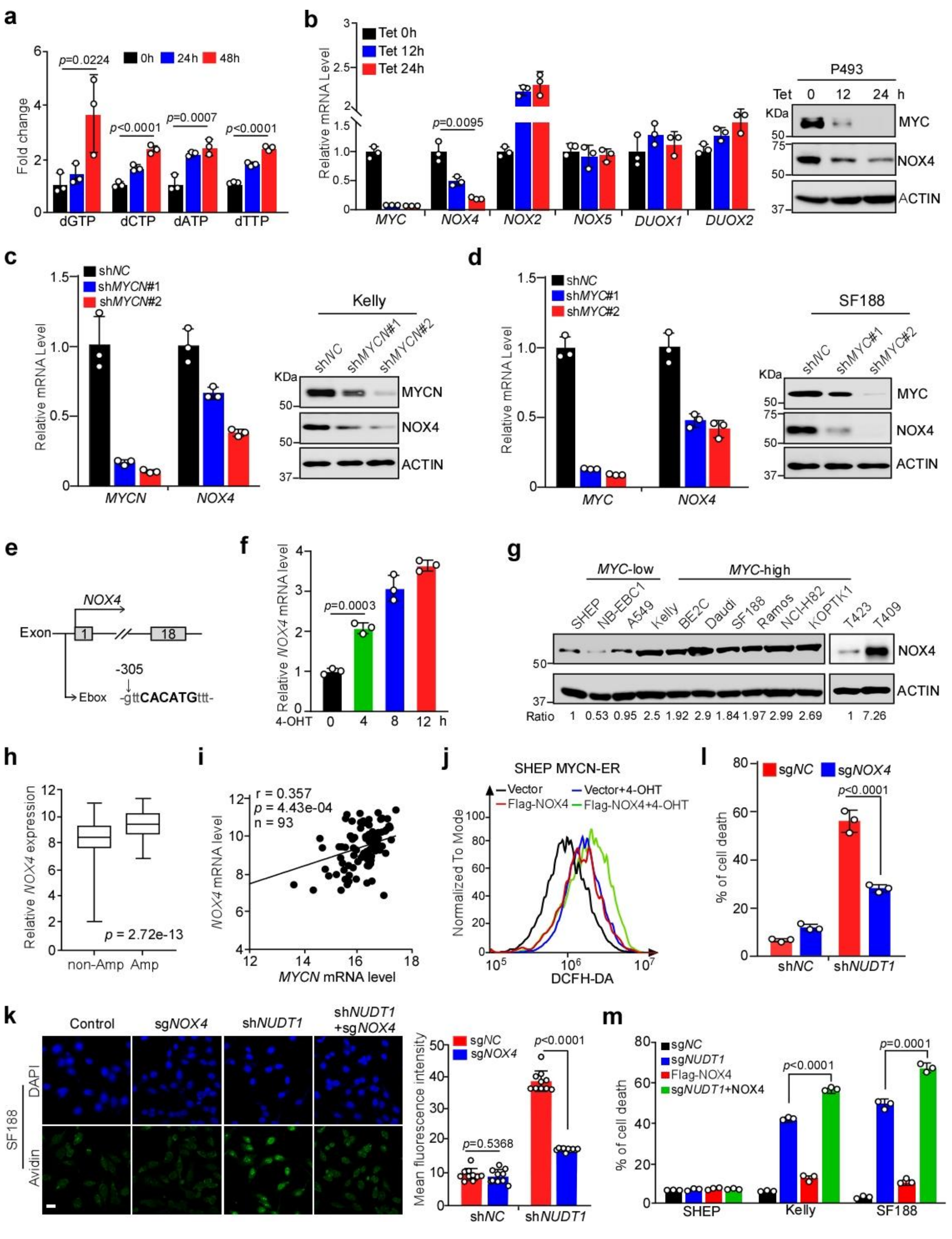

Supplementary Figure 2. MYC directly activates NOX4 transcription.

a Detection of nucleotide levels in SHEP MYCN-ER cells treated with 4-OHT for indicated times. Relative nucleotide levels are expressed as mean fold changes ± SD from technical triplicates. **b** qPCR analysis of *NOX* family members in P493 cells subjected to Tet exposure (100 ng/ml) for indicated times. Immunoblot of NOX4 is shown on the right, with β-Actin as a loading control. **c, d** *MYCN* or *MYC* was depleted by specific shRNAs in Kelly (**c**) or SF188 (**d**) cells. NOX4 expression was analyzed by qPCR and immunoblotting. **e** Schematic presentation of potential MYC binding site (E-box, from -302 to -296) on the NOX4 promoter. **f** Real-time qPCR analysis of *NOX4* in SHEP MYCN-ER cells upon 4-OHT treatment (200 nM). **g** Immunoblot of NOX4 in

tumor cells and tumor samples, with β-Actin as a loading control. This experiment was independently repeated three times with similar results. **h** Relative expression of *NOX4* in 643 primary neuroblastoma tumors (GSE45547). non-Amp, *MYCN*-nonamplified tumors (n = 550); Amp, *MYCN*-amplified tumors (n = 93). **i** Correlation between mRNA levels of *NOX4* versus *MYCN* in 93 *MYCN*-amplified primary neuroblastoma tumors. **j** Detection of ROS levels in SHEP MYCN-ER cells with or without NOX4 overexpression, treated with 4-OHT (200 nM) for 24 h. **k** Immunofluorescence images of 8-oxo-dGTP incorporation in DNA in SF188 cells expressing *NUDT1* shRNA and *NOX4* sgRNA. Scale bar, 20 μm. Quantifications of fluorescence signals are shown on the right (n=10 images). Data are means ± SD. **l** Cell death analysis of SF188 cells in (**k**). **m** Cell death analysis of indicated tumor cells overexpressing NOX4 and infected with *NUDT1* sgRNA. **b-d, f, l, m**: data are shown as averages of technical triplicates, and these experiments were independently repeated three times with similar results; **k**: this experiment was independently repeated twice with similar results. Statistical significance was determined by one-way ANOVA (**a, b, f**) or two-way ANOVA (**k, l, m**).

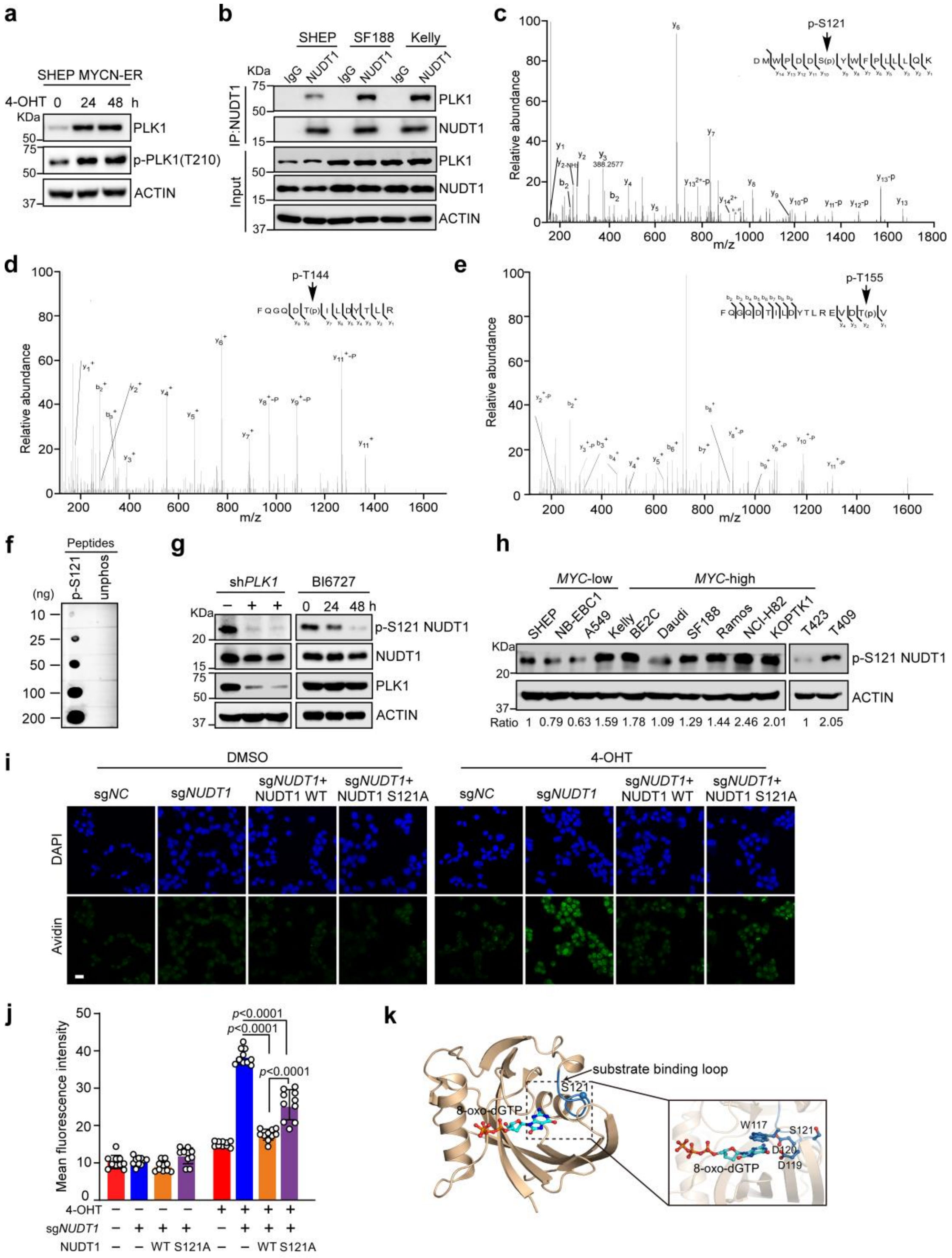

Supplementary Figure 3. PLK1 interacts with and phosphorylates NUDT1.

a Immunoblots of PLK1 and PLK1 p-T210 in SHEP MYCN-ER cells upon 4-OHT treatment (200 nM) for indicated time points. **b** Co-IP to detect interaction between endogenous NUDT1 and PLK1 in indicated cells. **c-e** Mass spectrometry spectrum of NUDT1 S121(**c**), T144(**d**) and T155(**e**) phosphorylation signals. **f** Dot blot analyzing the specificity of anti-NUDT1 pS121 antibody using synthesized phosphorylated (PDDSYWF) and unphosphorylated (unphos) peptide. **g** Immunoblots of NUDT1 p-S121 in SF188 cells transfected with PLK1 shRNA or exposed to BI6727 (20 nM). **h** Immunoblot of NUDT1 p-S121 in tumor cells and tumor samples,

with β-Actin as a loading control. **i** Immunofluorescence images of 8-oxo-dGTP in *NUDT1* depleted SHEP MYCN-ER cells expressing NUDT1 WT or the S121A mutant. Scale bar, 20 μm. **j** Quantification of 8-oxo-dGTP incorporation in SHEP MYCN-ER cells (n=10 images) in (**i**). Data are shown as means ± SD. **k** Cartoon representation of structure of the NUDT1 and 8-oxo-dGTP complex (PDB:5FSI). NUDT1, 8-oxo-dGTP and the substrate binding loop are colored in wheat, cyan, and skyblue, respectively. Residues involved in the interaction with 8-oxo-dGTP's guanine base are shown as stick-and-ball models. **a, b, g, h**: these experiments were independently repeated three times with similar results; **f**: this experiment was independently repeated twice with similar results. Statistical significance was determined by two-way ANOVA (**j**).

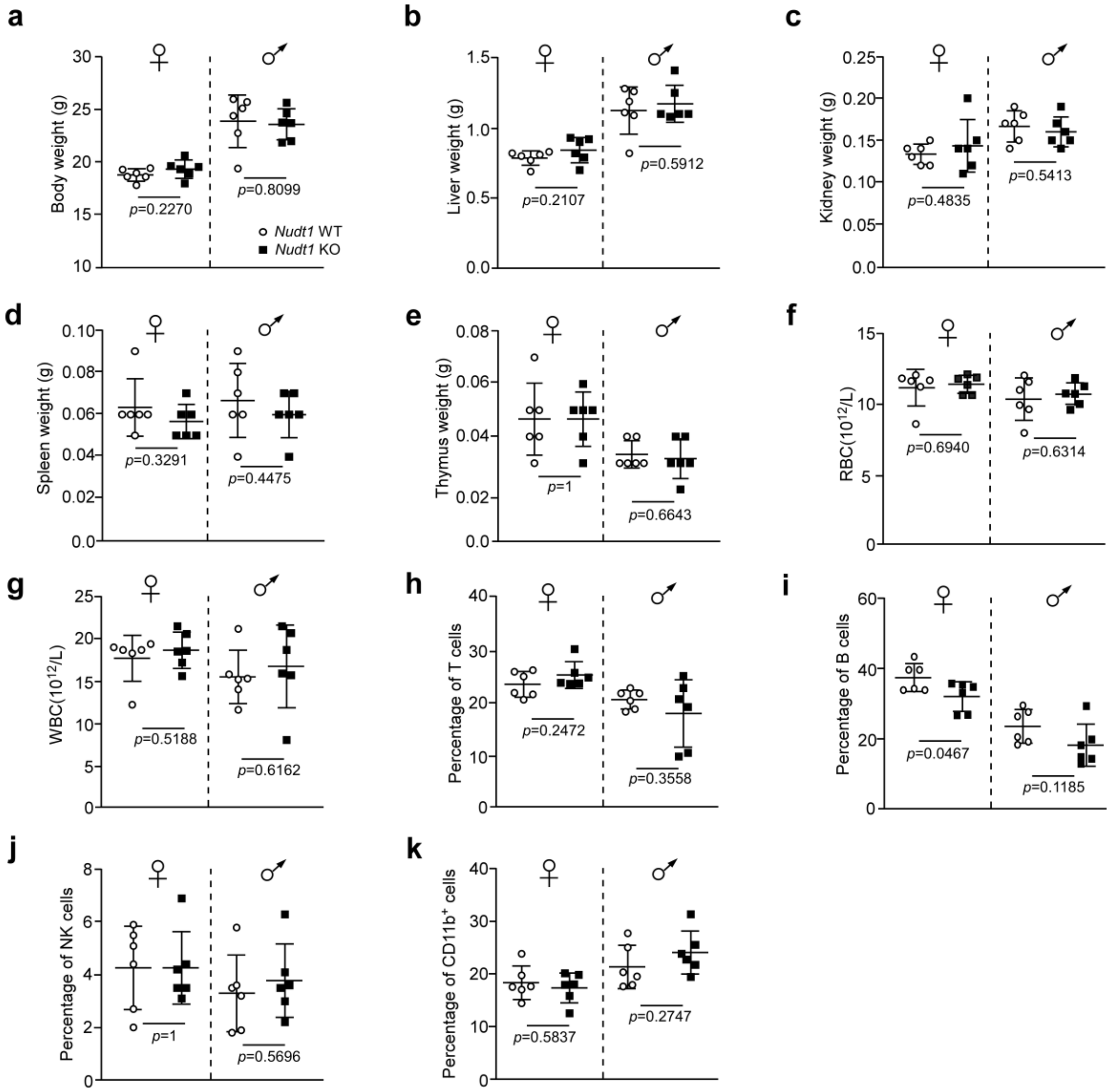

Supplementary Figure 4. Physiologic measurement of *Nudt1*$^{-/-}$ mice.

a Body weight of *Nudt1*$^{-/-}$ male and female mice and age-matched controls. **b-e** Assessment of indicated organ weights from *Nudt1*$^{-/-}$ male and female mice and age-matched controls. **f, g** Hematologic parameters of *Nudt1*$^{-/-}$ male and female mice and age-matched controls. **h-k** Percentages of T, B, NK and myeloid cells in *Nudt1*$^{-/-}$ male and female mice and age-matched controls. Above all, six mice were analyzed in each group. Unpaired two-tailed Student's *t*-test was used.

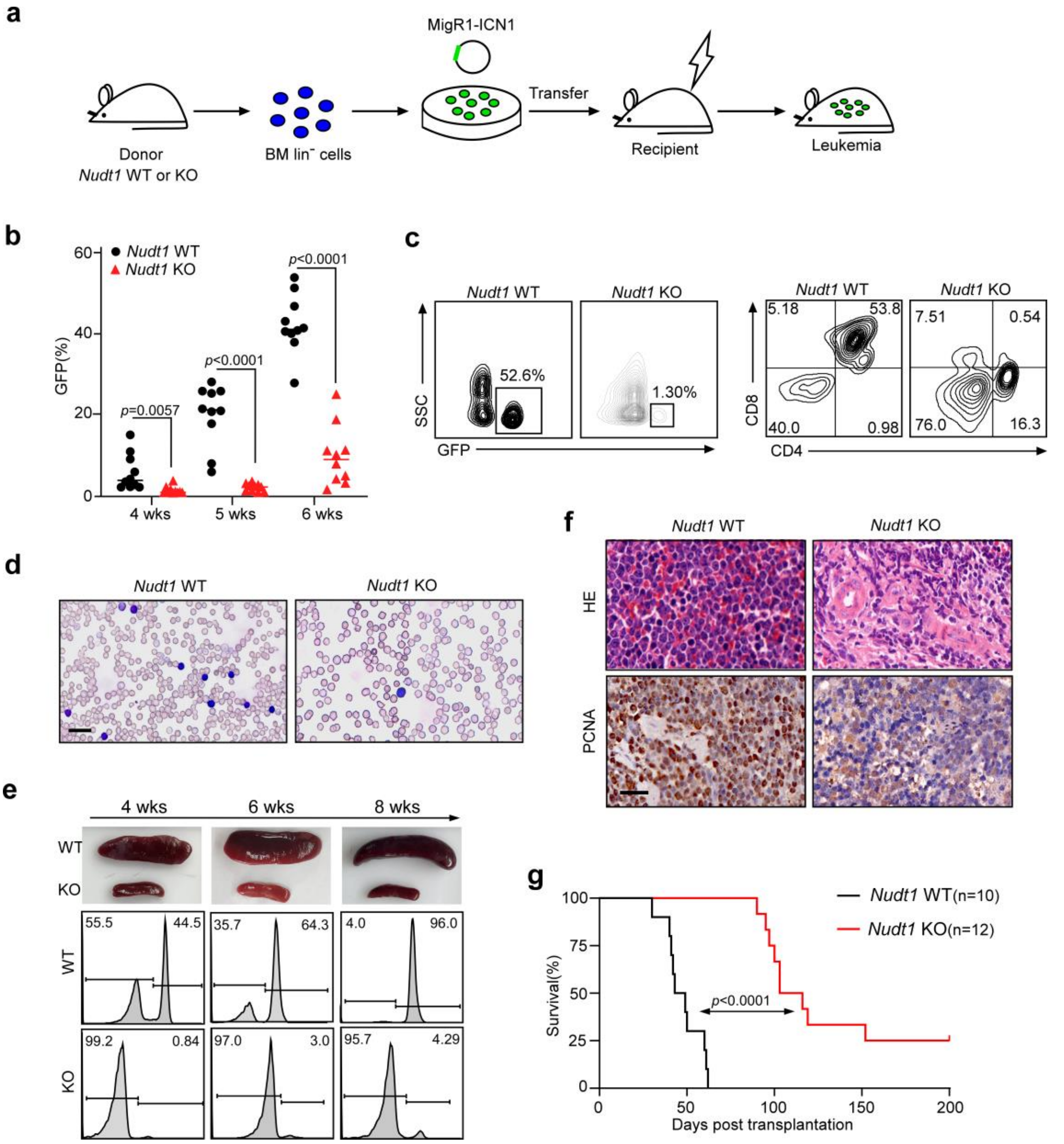

Supplementary Figure 5. *Nudt1* deficiency inhibits T-cell leukemogenesis.

a Graphical illustration of NOTCH1-induced T-ALL mouse model. Lin^- BM cells from $Nudt1^{+/+}$ or $Nudt1^{-/-}$ mice were infected with MigR1-ICN1 retroviruses and intravenously injected into irradiated recipient mice, followed by assessment of leukemia dissemination. **b** Determination the percentages of GFP^+ cells in peripheral blood (PB) at the indicated time points post transplantation on a flow cytometer (n=10 per group). Data are means ± SD. Statistical significance was determined by Unpaired two-tailed Student's *t*-test. **c** Representative flow cytometry analysis plot of GFP^+ (left) and $CD4^+CD8^+$ DP (right) leukemia cell distribution 6 weeks post transplantation. **d** Representative images of peripheral blood upon Wright-Giemsa staining. Scale bar, 25 µm. **e** Spleen images (upper) and flow cytometry analysis of GFP^+ leukemia cells (bottom) at the indicated time post transplantation. **f** Representative images of Hematoxylin & Eosin (H&E) (upper) and immunohistochemical staining for PCNA (bottom) from spleen. Scale bar, 50 µm. **g** Kaplan-Meier survival curves of ICN1-induced T-ALL mice using $Nudt1^{+/+}$ or $Nudt1^{-/-}$ as donor. Significance was determined by log-rank test.

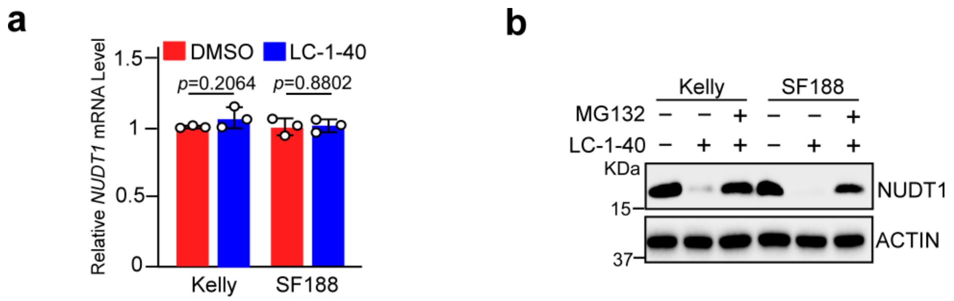

Supplementary Figure 6. LC-1-40 is a specific and potent NUDT1 degrader.
a qPCR analysis of NUDT1 in Kelly and SF188 cells upon LC-1-40 (50 nM) treatment. Data shown as average ± SD from technical replicates. Statistical significance was determined by unpaired two-tailed Student's *t*-test. **b** Immunoblot of NUDT1 in Kelly and SF188 cells with or without LC-1-40 treatment (50 nM), with β-Actin as a loading control. Cells were treated with MG132 (10 μM) for 6 h before harvest as indicated. All experiments were independently repeated three times with similar results.

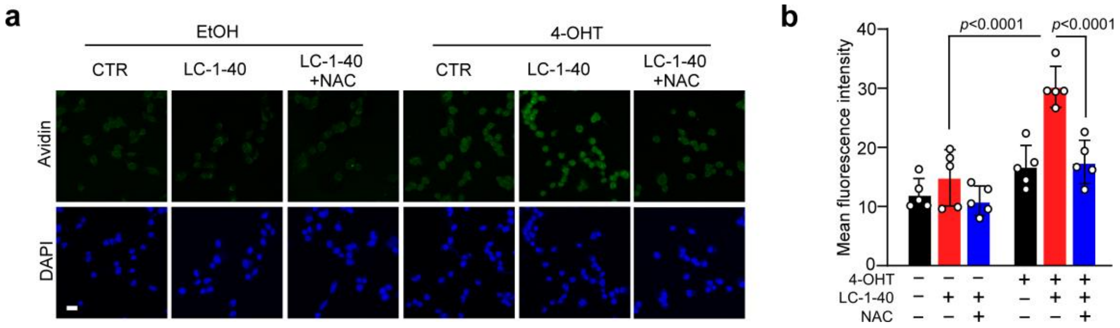

Supplementary Figure 7. NAC inhibited LC-1-40 induced nucleotide oxidation upon MYCN activation in SHEP cells.

a Immunofluorescence images of 8-oxo-dGTP production in SHEP MYCN-ER cells. Scale bar, 20 μm. **b** Quantifications of fluorescence signals (n=5 images). Data are shown as means ± SD. Statistical significance was determined by two-way ANOVA.

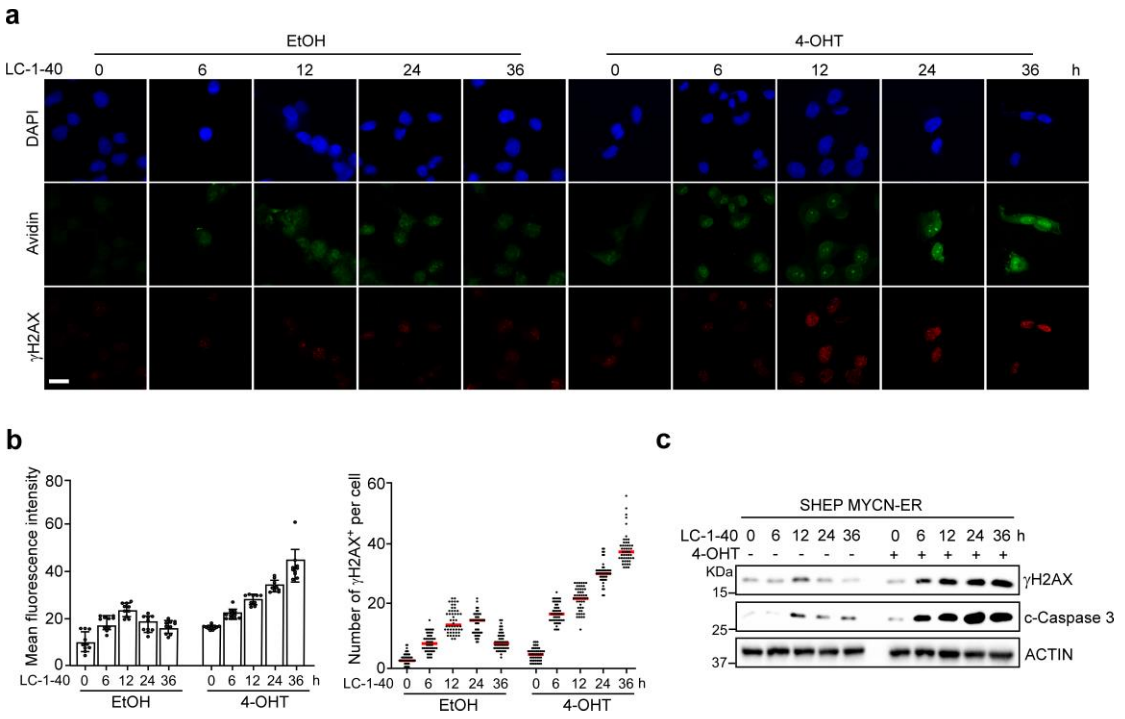

Supplementary Figure 8. LC-1-40 induced lethal 8-oxo-dGTP and γH2AX accumulation in 4-OHT treated SHEP MYCN-ER cells.

a Immunofluorescence images of 8-oxo-dGTP and γH2AX in SHEP cells with the indicated treatment. Scale bar, 20 μm. Quantifications of 8-oxo-dGTP fluorescence signals (n=10 images) and γH2AX foci per cell (n=50 cells) are shown in **b**. **c** Immunoblots of γH2AX and c-Caspase 3 in SHEP MYCN-ER cells subjected to the indicated treatment, with β-Actin as a loading control. This experiment was independently repeated three times with similar results.

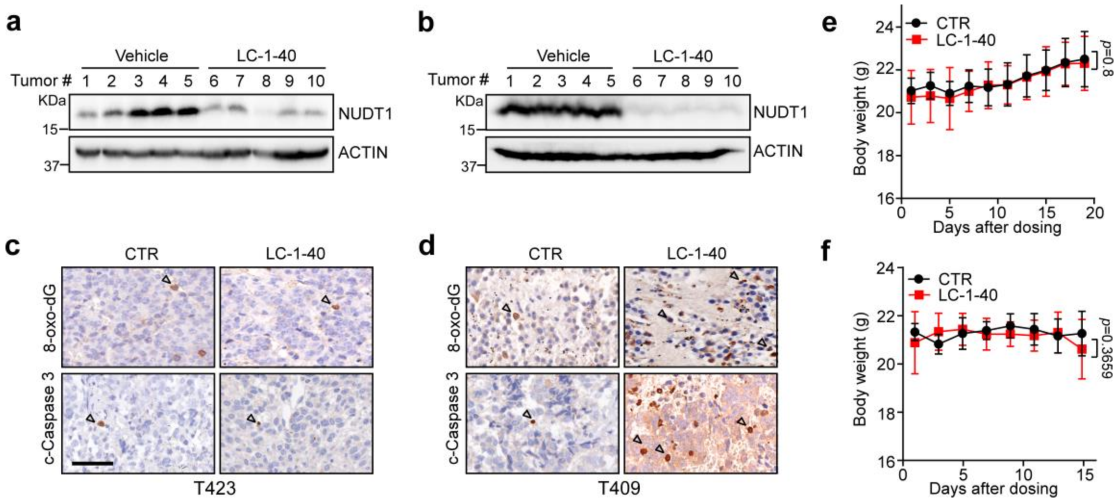

Supplementary Figure 9. LC-1-40 causes more oxidized nucleotides in MYC-driven tumors.

a, b Immunoblots of NUDT1 from T423 (**a**) and T409 xenografts tumors (**b**). 5 tumors were analyzed per group. **c, d** Representative histological images of 8-oxo-dGTP and c-Caspase 3 staining from one of the paraffin-embedded tumor sections (**c,** T423) and (**d,** T409). Triangles denote positive-stained cells. Scale bar, 50 µm. **e, f** Body weights of mice in Figure 6f-6i at days post injection with vehicle or LC-1-40. Data are shown as averages ± SD (n = 5 mice per group). Statistical significance was determined by two-way ANOVA (**e, f**).

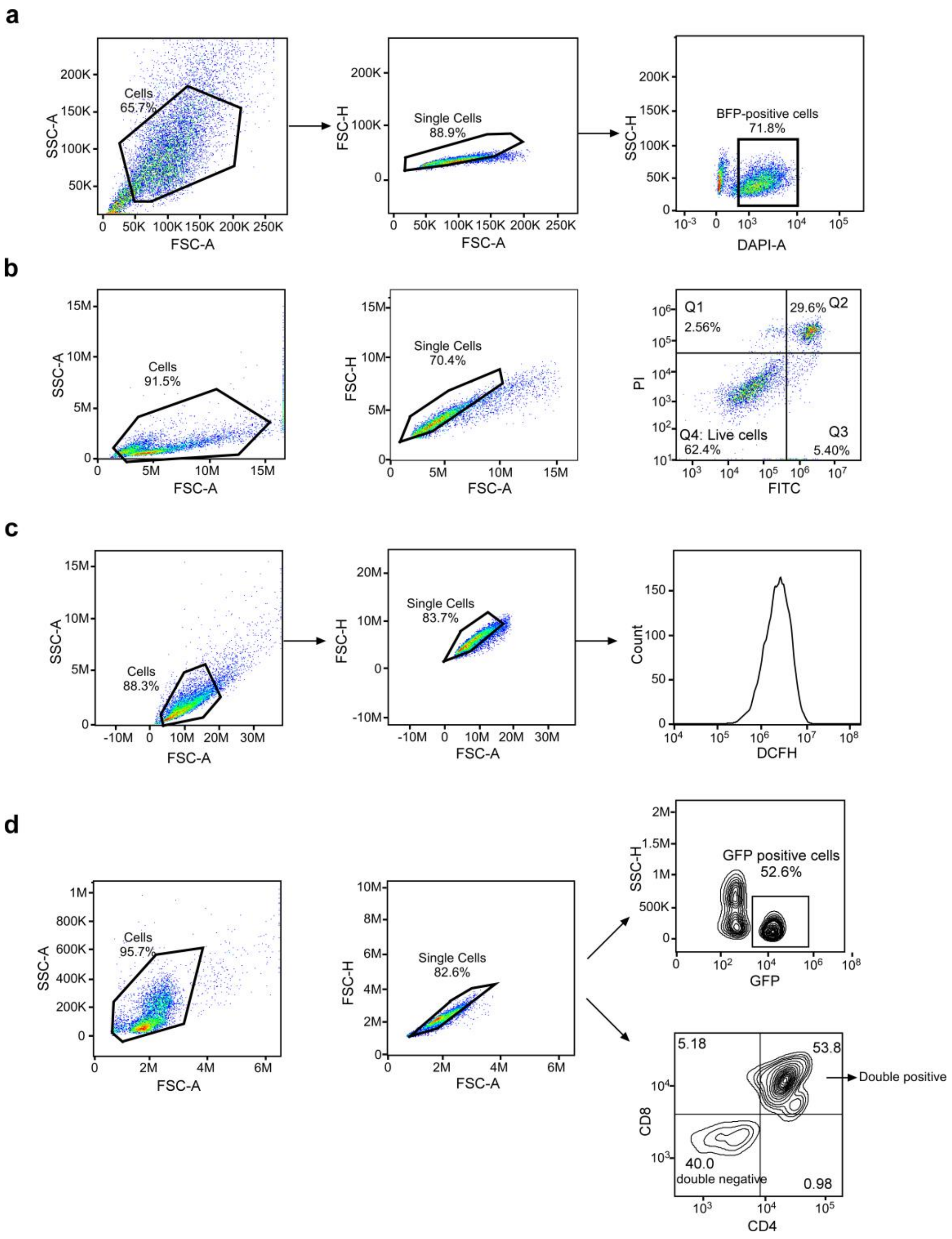

Supplementary Figure 10. Sorting and gating strategies.

a Representative sorting strategy for BFP-Cas9 positive cell in Figure 1a. **b** Representative gating strategy for cell death analysis. **c** Representative gating strategy for ROS detection in Figure 2a, 2d and Supplementary Figure 2j. Statistical analysis of defined cells are shown. **d** Representative gating strategy for GFP^+ and $CD4^+CD8^+$ (Double positive) leukemia cells analysis in Supplementary Figure 5c. Statistical analysis of defined cells are shown.

Supplementary Table 1. Primary NB samples information

Sample	Gender	Age	Metastasis	Risk group	*MYCN* status
T423	Female	~ 9 years	Yes	High	Non-amplified
T409	Male	~ 4 years	Yes	High	Amplified

Supplementary Table 2. Primers used in the study

**Primers for qRT-PCR**	
EIF4E forward	5'-AACAAACGGGGAGGACGATG-3'
EIF4E reverse	5'-CCAAAAGCGATCGAGGTCAC-3'
ODC1 forward	5'-CCTTTTGGAACGGGCGAAAG-3'
ODC1 reverse	5'-CAAAAACACAGCGGGCATCA-3'
NCL forward	5'-GCTTCAGCGGCTTTAGGACA-3'
NCL reverse	5'-TGGAAACACGCACTAAGCCA-3'
NOX2 forward	5'-CTGCTCAACAAGAGTTCGAAGA-3'
NOX2 reverse	5'-GCCTCCTTCAGGGTTCTTTATT-3'
NOX4 forward	5'-GACTTTACAGGTATATCCGGAGCAA-3'
NOX4 reverse	5'-TGCAGATACACTGGACAATGTAGA-3'
NOX5 forward	5'-CTATTGGACTCACCTGTCCTACC-3'
NOX5 reverse	5'-GGAAAAACAAGATTCCAGGCAC-3'
DUOX1 forward	5'-GGCTTGTCTCCCATTACCC-3'
DUOX1 reverse	5'-GCCCATTCCTGGTGTTCTC-3'
DUOX2 forward	5'-TCCAGAAGGCGCTGAACAG-3'
DUOX2 reverse	5'-GCGACCAAAGTGGGTGATG-3'
MYCN forward	5'-CCACAAGGCCCTCAGTACC-3'
MYCN reverse	5'-TCTTCCTCTTCATCATCTTCATCA-3'
c-MYC forward	5'-CTGGTGCTCCATGAGGAGA-3'
c-MYC reverse	5'-CCTGCCTCTTTTCCACAGAA-3'
ACTIN forward	5'-CATGTACGTTGCTATCCAGGC-3'
ACTIN reverse	5'-CTCCTTAATGTCACGCACGAT-3'
18S forward	5'-CAGCCACCCGAGATTGAGCA-3'
18S reverse	5'-TAGTAGCGACGGGCGGTGTG-3'
NUDT1 forward	5'-GTGCAGAACCCAGGGACCAT-3'
NUDT1 reverse	5'-GCCCACGAACTCAAACACGA-3'
**Primers for ChIP assay**	
ACTIN promoter forward	5'-GACTTCTAAGTGGCCGCAAG-3'
ACTIN promoter reverse	5'-TTGCCGACTTCAGAGCAAC-3'
NOX4 promoter forward	5'-CCGGCTCAAATTTCGTTAC-3'
NOX4 promoter reverse	5'-GCCTGTTGTTGTGGCTG-3'
**Primers for genotyping**	
NUDT1 WT-F	5'-AGTGTCTGAAGACAGCTACAGTATATAA-3'
NUDT1 WT-R	5'-GACCCTCCTAAGAGGCTGCAGTGTA-3'

NUDT1 MUT-R	5’-AGCCCTCCTTTGGGCTTTCCTTA-3’
TH-MYCN OUT1	5’-TTGGCACACACAAATGTATATACACAATGG-3’
TH-MYCN Chr18F1	5’-ACTAATTCTCCTCTCTCTGCCAGTATTTGC-3’
TH-MYCN Chr18F2	5’-TGCCTTATCCAAAATATAAATGCCCAGCAG-3’
**sgRNA**	
Ctrl sgRNA	5’-GTAGTCGGTACGTGACTCGT-3’
NUDT1 sgRNA	5’-GCAAGAAGGAGAGACCATCG-3’
NOX4 sgRNA	5’-GAGGTTAAGAACAGATGCTG-3’
**shRNA**	
shGFP	5’-GCGCGATAGCGCTAATAATTT-3’
NUDT1 shRNA	5’-CCTGCTTCAGAAGAAGAAATT-3’
MYCN sh#1	5’-GCCAGTATTAGACTGGAAGTT-3’
MYCN sh#2	5’-CACCTCCATGACAGCGCTAAA-3’
MYC sh#1	5’-CAGTTGAAACACAAACTTGAA-3’
MYC sh#2	5’-CCTGAGACAGATCAGCAACAA-3’
PLK1 sh#1	5’-CGATACTACCTACGGCAAATT-3’
PLK1 sh#2	5’-CGCCTCATCCTCTACAATGAT-3’
