# Supplementary material for: Therapeutic targeting nudix hydrolase 1 creates a MYC-driven metabolic vulnerability
Source: Nat Commun. 2024 Mar 16;15:2377. doi: 10.1038/s41467-024-46572-6 (PMC10944511; doi:10.1038/s41467-024-46572-6)
Supplement: Supplementary file 4 — Description of Additional Supplementary Files [file 41467_2024_46572_MOESM4_ESM.pdf]

Description of Additional Supplementary Files

File Name: Supplementary Data 1

Description: Gene lists identified exhibiting >2-fold reduction ($p < 0.05$) in 4-OHT treated cells.

File Name: Supplementary Data 2

Description: A list of all sgRNA sequences used in sgRNA library

File Name: Supplementary Data 3

Description: A list of NUDT1-interacting proteins in 4-OHT treated group with p value < 0.05 and a fold change >2
